# Supplementary material for: The influence of hip muscle strength on gait in individuals with a unilateral transfemoral amputation
Source: PLoS One. 2020 Sep 2;15(9):e0238093. doi: 10.1371/journal.pone.0238093 (PMC7467296; doi:10.1371/journal.pone.0238093)
Supplement: S1 Table — # = nothing specified. (DOCX) [file pone.0238093.s001.docx]

Prior to data collection they were asked about their current health status and sports activities, which are compiled in S1 Table .

**S1 Table: Self-reported health status and sports activities of the participants with TFA**

| Person with TFA | self-reported comorbidities | self-reported sports activities |
| --- | --- | --- |
| 1 | # | # |
| 2 | # | # |
| 3 | # | cycling |
| 4 | # | cycling, table tennis, skiing |
| 5 | # | cycling |
| 6 | high blood pressure | athletics, strength training |
| 7 | drop foot sound side | # |
| 8 | # | # |
| 9 | # | nordic walking, swimming |
| 10 | # | strength training, cycling |
| 11 | # | swimming |
| 12 | # | # |
| 13 | # | hiking, cycling |

# = nothing specified
