# Supplementary material for: The influence of hip muscle strength on gait in individuals with a unilateral transfemoral amputation
Source: PLoS One. 2020 Sep 2;15(9):e0238093. doi: 10.1371/journal.pone.0238093 (PMC7467296; doi:10.1371/journal.pone.0238093)
Supplement: S2 Table — Values Spearman's rho with its corresponding p-value; level of significance was set to p < .0036; * = statistical significant correlation; # = moderate, + = strong, ! = very strong; correlations with a Spearman’s ρ of 0.40–0.59 were classified as moderate, 0.60–0.79 as strong, and 0.80–1.0 as very strong correlations). (DOCX) [file pone.0238093.s002.docx]

**S2 Table:** Correlations of sagittal and coronal plane gait parameters with their corresponding maximum isometric moments (MIM) of the hip.

|  |  |  | **Participants** | | | |
| --- | --- | --- | --- | --- | --- | --- |
|  |  |  | **with trans femoral amputation (TFA)** | | **without  impairment (REF)** | |
|  |  |  | Hip MIM (N m/kg) | | Hip MIM (N m/kg) | |
|  |  |  | ext. | flex. | ext. | flex. |
| **correlations sagittal plane  (Spearman's rho)** | Hip MIM (N m/kg) | ext. | 1 | **0.825!** | 1 | **0.818!** |
|  |  |  | - | **p = 0.001*** | - | **p < 0.001*** |
|  |  | flex. | **0.825!** | 1 | **0.818!** | 1 |
|  |  |  | **p = 0.001*** | - | **p < 0.001*** | - |
|  | CGA maximum hipmoments in stance (N m/kg) | ext. | 0.042 | 0.371 | -0.216 | -0.130 |
|  |  |  | p = 0.90 | p = 0.23 | p = 0.39 | p = 0.61 |
|  |  | flex. | 0.235 | 0.490# | 0.253 | -0.045 |
|  |  |  | p = 0.46 | p = 0.11 | p = 0.31 | p = 0.86 |
|  | CGA kinematics hip pelvis & trunk ranges 0-100%  gait cycle (in degrees) | hip flex. & ext. | 0.025 | -0.144 | -0.188 | -0.438# |
|  |  |  | p = 0.94 | p = 0.66 | p = 0.45 | p = 0.07 |
|  |  | pelvic tilt | -0.389 | -0.123 | -0.085 | -0.235 |
|  |  |  | p = 0.21 | p = 0.70 | p = 0.74 | p = 0.35 |
|  |  | trunk tilt | 0.053 | -0.144 | 0.276 | 0.362 |
|  |  |  | p = 0.87 | p = 0.66 | p = 0.27 | p = 0.14 |
|  |  |  |  |  |  |  |
|  |  |  | Hip MIM (N m/kg) | | Hip MIM (N m/kg) | |
|  |  |  | abd. | add. | abd | add. |
| **correlations coronal plane (Spearman's rho)** | Hip MIM (N m/kg) | abd. | 1 | 0.661+ | 1 | **0.763+** |
|  |  |  | - | p = 0.019 | - | **p < 0.001*** |
|  |  | add. | 0.661+ | 1 | **0.763+** | 1 |
|  |  |  | p = 0.019 | - | **p < 0.001*** | - |
|  | CGA maximum hipmoments in stance (N m/kg) | abd. | -0.032 | 0.053 | -0.649+ | -0.592# |
|  |  |  | p = 0.92 | p = 0.87 | p = 0.004 | p = 0.01 |
|  |  | add. | -0.421# | -0.628+ | 0.217 | 0.037 |
|  |  |  | p = 0.17 | p = 0.03 | p = 0.39 | p = 0.88 |
|  | CGA kinematics hip pelvis & trunk ranges 0-100%  gait cycle (in degrees) | hip abd. & add. | -0.123 | -0.406# | -0.603+ | -0.629+ |
|  |  |  | p = 0.70 | p = 0.19 | p = 0.01 | p = 0.01 |
|  |  | pelvic obliquity | -0.032 | -0.242 | -0.486# | -0.446# |
|  |  |  | p = 0.92 | p = 0.45 | p = 0.04 | p = 0.06 |
|  |  | trunk obliquity | -0.452# | -0.203 | 0.557# | 0.201 |
|  |  |  | p = 0.14 | p = 0.53 | p = 0.02 | p = 0.42 |

Values Spearman's rho with its corresponding p-value; level of significance was set to p<.0036; *= statistical significant correlation; #=moderate, + = strong, ! = very strong; correlations with a Spearman’s ρ of 0.40-0.59 were classified as moderate, 0.60-0.79 as strong, and 0.80-1.0 as very strong correlations)
