# Supplementary figures and images for: The influence of hip muscle strength on gait in individuals with a unilateral transfemoral amputation
Source: PLoS One. 2020 Sep 2;15(9):e0238093. doi: 10.1371/journal.pone.0238093 (PMC7467296; doi:10.1371/journal.pone.0238093)

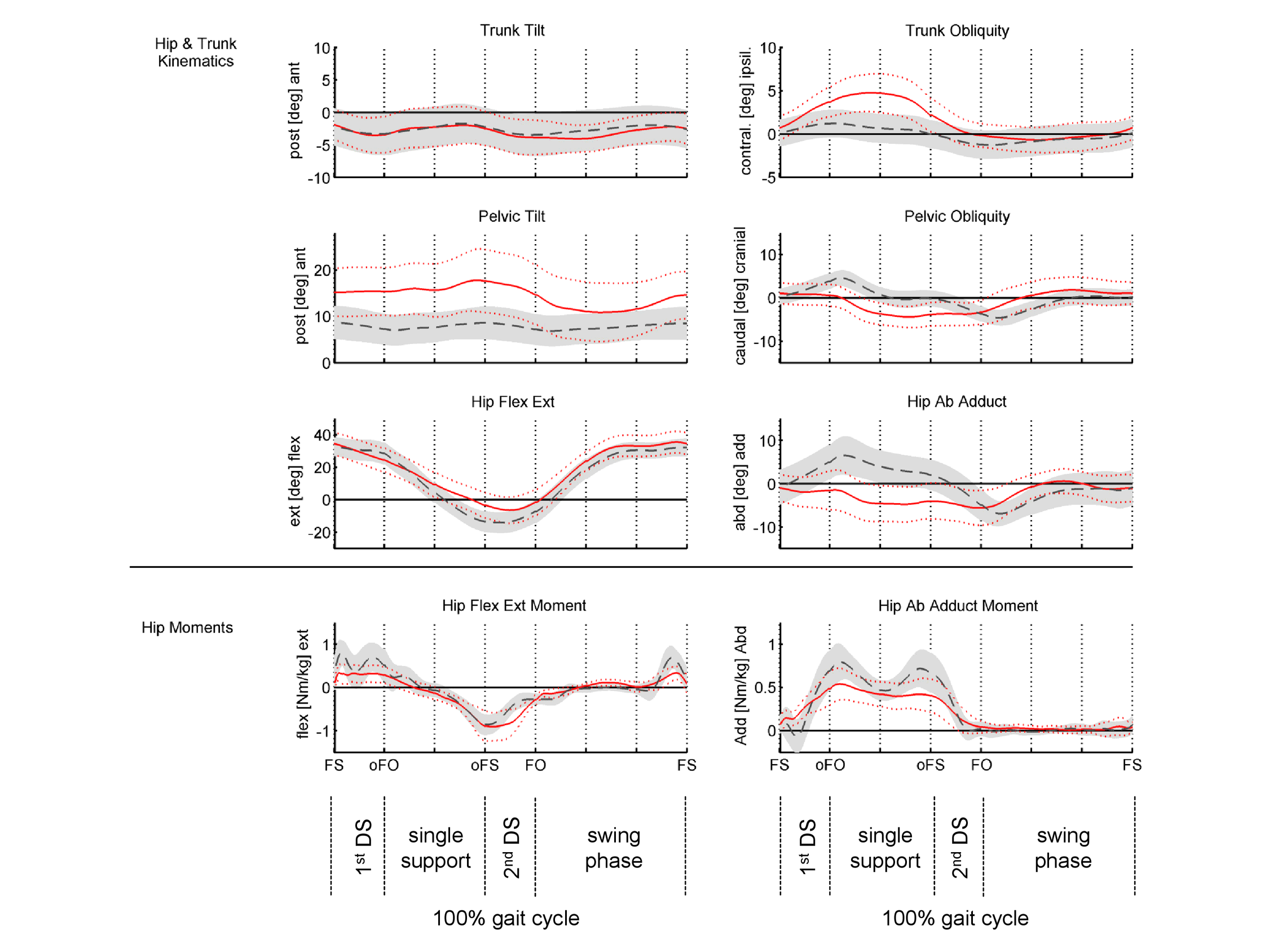

Supplement: S1 Fig — Red, solid line = mean kinematics and kinetics during walking of participants with TFA walking @ 1.22 (±0.22) m/s; red, dashed line = standard deviation of the TFA mean; grey, dashed line = mean moments during walking of REF @ 1.44(±0.17) m/s; grey band = standard deviation of the REF mean; FS = foot strike; oFO opposite foot of; oFS = opposite foot strike; FO = foot off; DS = double support. (TIF) [file pone.0238093.s003.tif]

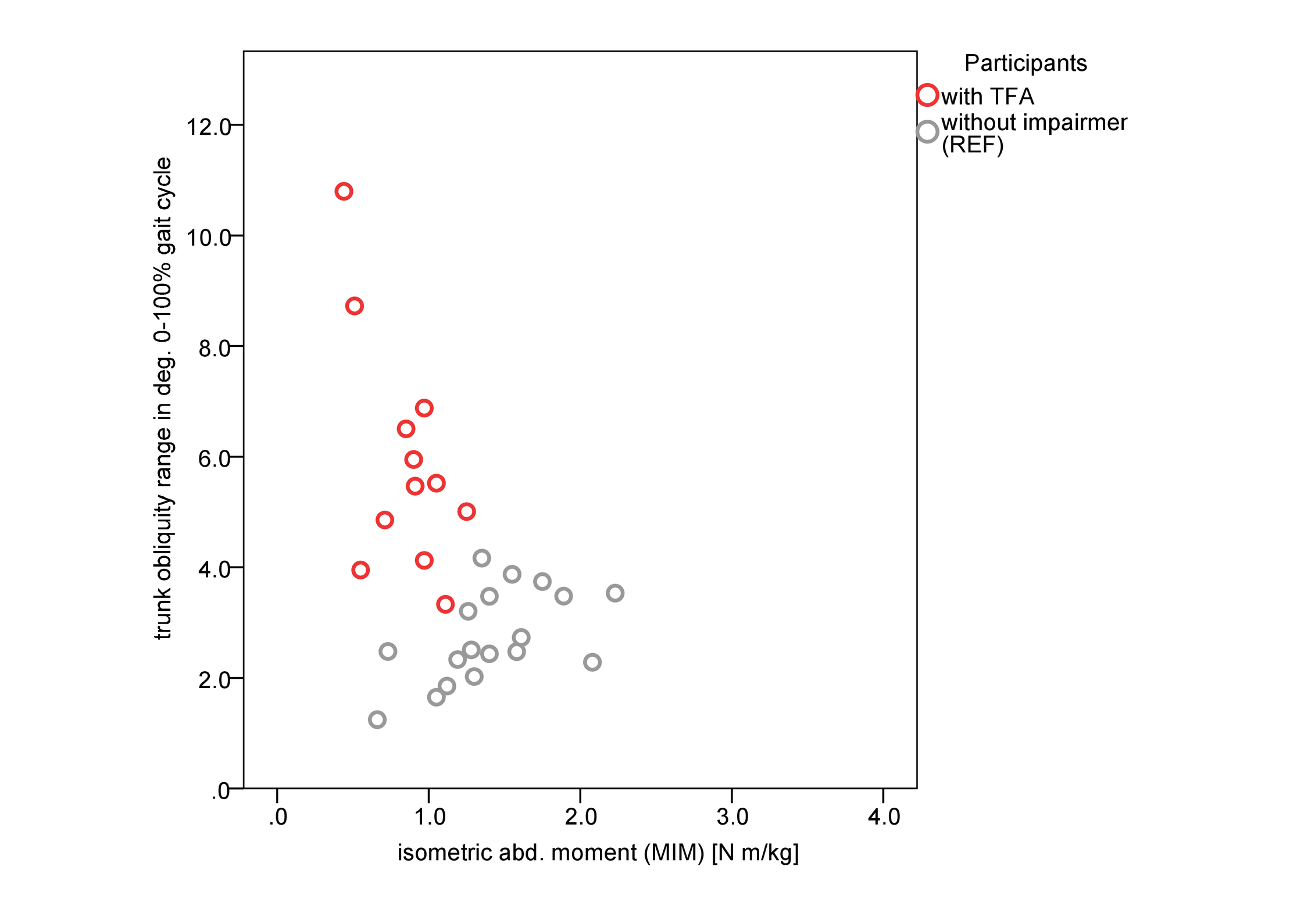

Supplement: S2 Fig — (TIF) [file pone.0238093.s004.tif]

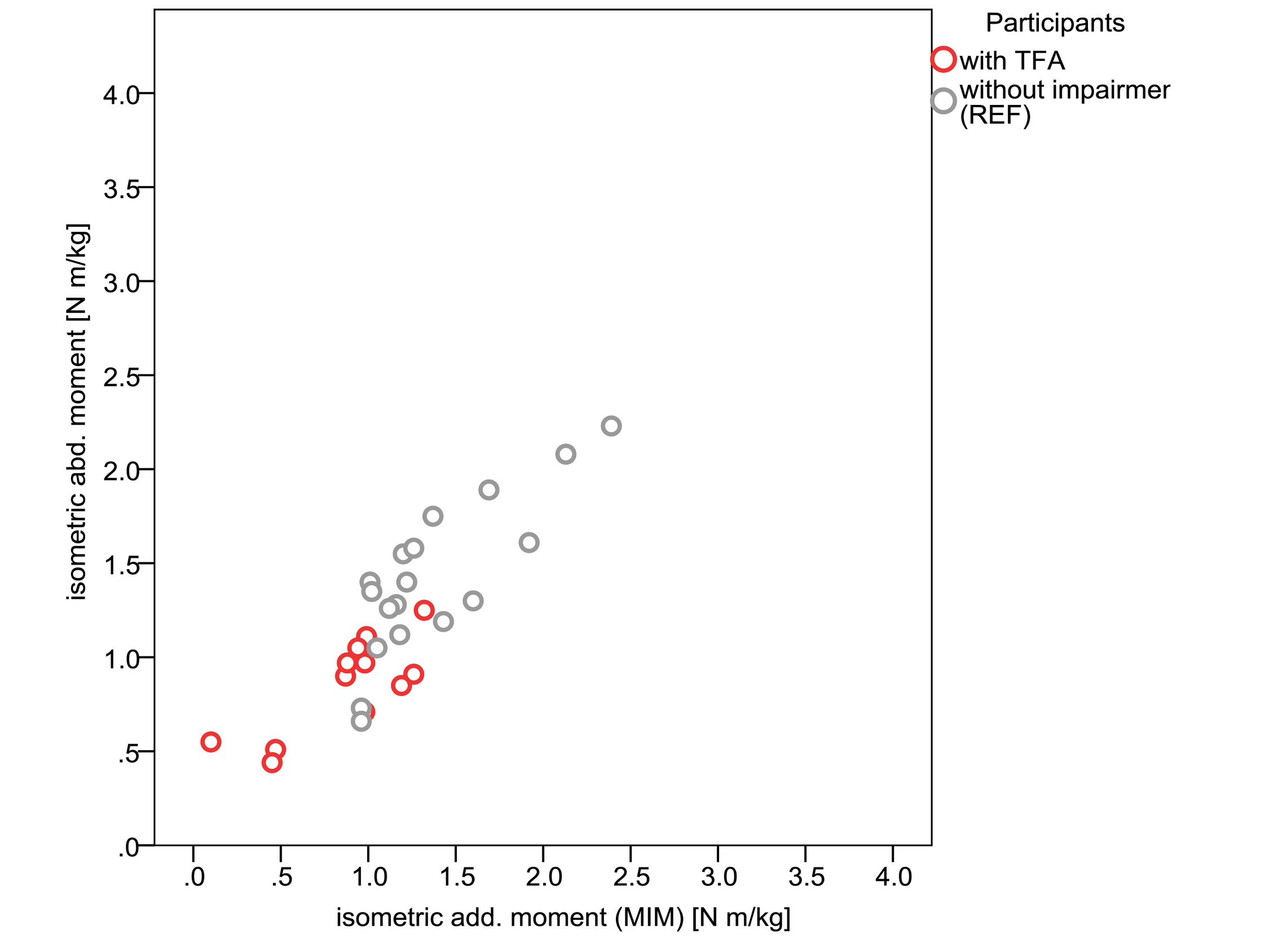

Supplement: S3 Fig — (TIF) [file pone.0238093.s005.tif]

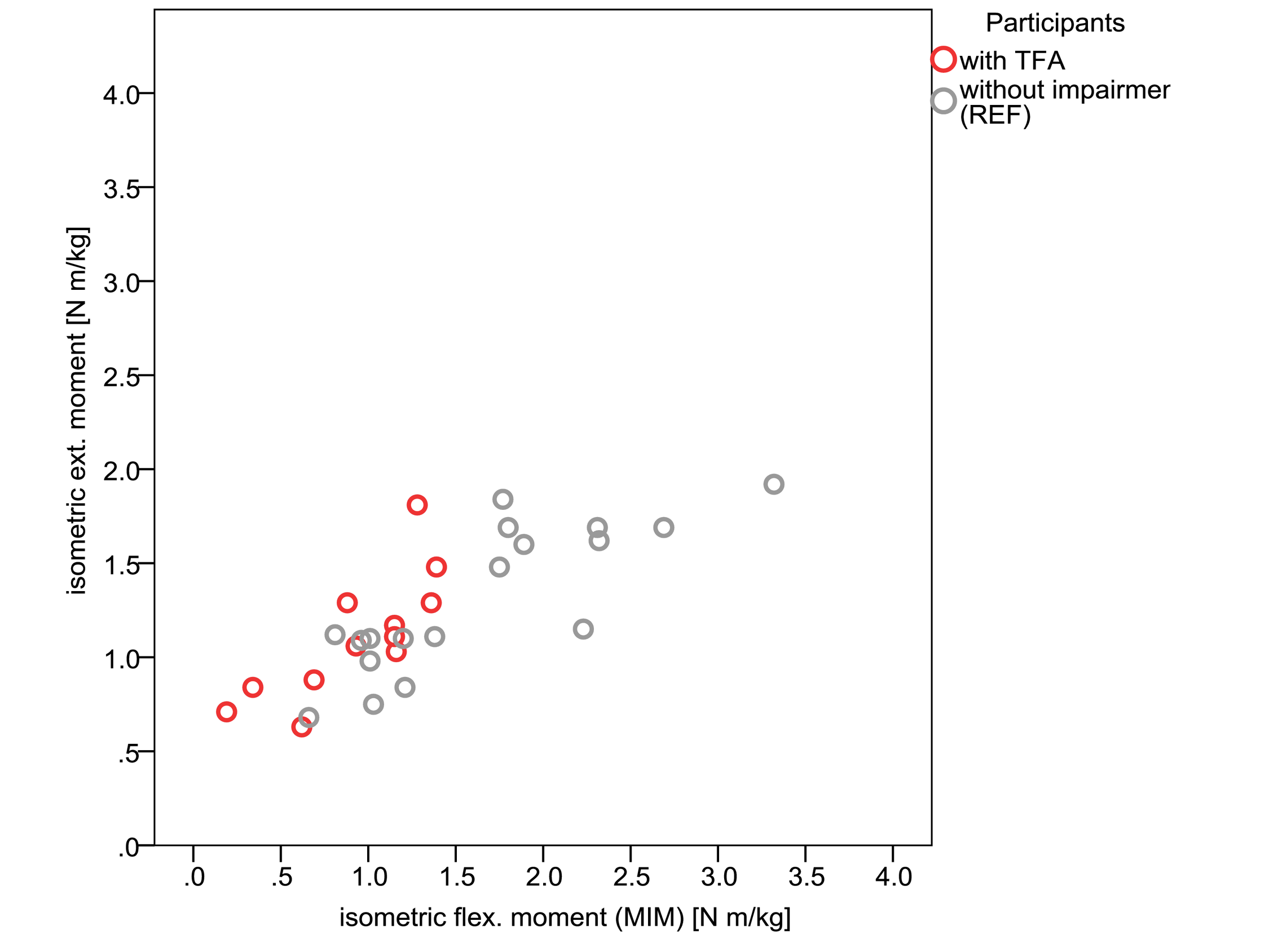

Supplement: S4 Fig — (TIF) [file pone.0238093.s006.tif]
