# Supplementary material for: The influence of hip muscle strength on gait in individuals with a unilateral transfemoral amputation
Source: PLoS One. 2020 Sep 2;15(9):e0238093. doi: 10.1371/journal.pone.0238093 (PMC7467296; doi:10.1371/journal.pone.0238093)
Supplement: Attachment — Submitted filename: TFA_strength_gait_2019.pdf [file pone.0238093.s007.pdf]

This manuscript investigates potential correlations of strength gait deviations, as well as compared a cohort of people with transfemoral amputation to people without amputation. The topic of this article is interesting because if we could see a gait deviation and then address it with rehabilitation (e.g., strength or balance), it would help people with amputation walk with less deviations. I have a series of comments below that reference major and minor concerns, and I have highlighted some of them with line numbers but did not highlight every instance that is repeated throughout the manuscript.

General questions: Is maximal strength important with over ground preferred walking speed, or is it more important to show how much of their overall capacity they need to use to walk compared with non-amputees? Is there a greater reserve strength for non-amputees? Maybe a deviation is chosen to save available muscle fibers and reserve capacity to respond to perturbations or minimize energy expenditure.

Major Comments

Abstract:

Line 51: Is this a prospective study? A prospective study identifies a population and then follows them and tries to make predictions about what will happen, whereas here you are trying to make correlations between gait deviations and strength deficits.

Lines 51-53: Aside from the prospective study comment, I like this statement because it is clear about what you did in this study.

Introduction:

Line 95: This is something done many times throughout the manuscript, and may just be a personal preference on my part, but other authors should be cited while their findings are prominent. "Probsting…" sentence adds nothing. Then line 104, "Ostchega et al…." did not prove a relationship, the results or findings of a study show something, the authors just write it up. Also, there are very few proven relationships, there is more evidence for some things than others, but one study (and multiple studies) does not prove a relationship.

Line 102: Unless you plan on talking about other forms of mobility, such as transfers, just say "this relates to walking."

Throughout the introduction there could be more standalone paragraphs, and rather than listing findings from study after study, the information could be synthesized or summarized. This relates to my comment above about authors not showing anything, but the overall data collected from many studies.

Lines 173 and 174: There should not be methods in the introduction.

Lines 182-183: Should hypothesized directionality of correlations, not just state there will be correlations.

Methods:

Line 213-215: I am not sure if participant 8 should be included in the data analysis, especially since he is a K2 ambulator and then he walked without crutches during his motion analysis. Walking without crutches is not his normal or preferred, and therefore the gait speed and mechanics are not preferred, whereas the participants were using preferred mechanics.

Lines 267-268: Did you standardize the amount of test trials for participants to try the OpTIMo?

Lines 284: General questions about OpTIMo, how widespread is this device clinically or in research? Is it a research-only device? How does it compare to typical clinical force dynamometry measures with patients laying down?

Line 287: What makes a trial the best?

Line 295: Why not look at the sound side too? Could be interesting when comparing to affected side and unaffected participants.

Statistics look right.

Results:

Line 310-311: All the differences were significant except for the step length.

Figure 3: Should label x and y axes rather than leave that to the title. Also, I do not know what FS, FO, oFS, and oFO are, I assume they are foot strike and foot off, but do not know.

Lines 342-345: What does negative and positive correlations mean in your results?

Discussion:

General comment: Should not repeat results, especially with p-values, in the discussion section, they should be in the results section alone.

Line 375: Remove distinguished, that is your spin and unnecessary.

Line 382: The results from Cappozzo agree with your results, do not confirm your results, and if anything, your results confirm their finding.

Lines 409-421: Listing many results in the discussion section, could be synthesized with the literature better.

Minor Comments

Abstract:

Line 47: Awkward wording “on the person concerned”

Line 59: should be “Kinematics” not “Kinematic”

Line 59: Could say determined, not derived, unless you derived equations.

Line 72: Could say “were identified, i.e. significantly lower speed,…”

Introduction:

Line 91: Could say “body integrity, perception…”

Line 98: “clear atrophy” does not make sense, atrophy alone works

Line 98: I recommend saying amputated or affected side, rather than ipsilateral side. I understand ipsilateral but I am not sure every reader will, it is more clear to say something like amputated, affected or involved.

Line 102: “particularly” does not add anything, like most adverbs

Line 126: What do you mean by “redundant”?

Line 127: Could say “Additionally, the…”

Line 128: What do you mean by “finite coupling”?

Line 130: Could say “Gholizadeh et al. underline the problems in…”

Line 138: Could say “Additionally, new…”

Line 164: Lower case i in ischial

Methods:

Line 193: clinical gait analysis can be replaced with CGA because the acronym is already listed in the beginning

Line 225: Is the L.A.S.A.R. posture device widely known? I am not sure if it should be explained more or you could stop the sentence with “recommendations.”

Line 240: What was the collection rate for the force plates?

Results:

Line 315: “in the patients” is awkward wording

Lines 320-321: “For CGA…the patient group.” is not a clear sentence, could be written better.

Line 325: Why is this schematic “exemplary”?

In table 2, Kg should be kg, and it should be N m and not Nm because they are 2 separate words.

Line 339: Should say “with TFA hip MIM of…” and not “with TFA Hip MIMof…”

Discussion:

Line 390: pattern, not patter

Line 445: Say For example, no E.g. to lead a sentence

Line 445: does rather than do

Line 449-452: Not a clear sentence.

Line 452: stabilize rather than stabile

Line 501: address rather than tackle deficits, tackle is in American football or Rugby rather deficits of our patients/clients
