# Supplementary material for: The influence of hip muscle strength on gait in individuals with a unilateral transfemoral amputation
Source: PLoS One. 2020 Sep 2;15(9):e0238093. doi: 10.1371/journal.pone.0238093 (PMC7467296; doi:10.1371/journal.pone.0238093)
Supplement: Attachment — Submitted filename: Response to Reviewers.docx [file pone.0238093.s009.docx]

Reviewer #1: The revised manuscript has been extensively rewritten in response to the reviewers’ comments. The literature in the introduction is now presented in a more synthesized form, the statistical analyses have been revised to account for multiple comparisons, and the discussion has largely been re-written to better relate the results to the literature and discuss the impact of potential confounds. In doing so, the clarity of the manuscript has been improved. A few minor issues remain, which should be addressed prior to publication.

Response to the Reviewer: We would like to thank the reviewer for the encouraging words and the many valuable suggestions. This was more than helpful and we appreciate the time and effort the reviewer put into their response.

The methods indicate that Spearman’s rank correlation was used, but in some places the results refer to “linear correlations” (e.g., Line 505 and S2_Figure). If the references to a linear correlation are correct, please include in the methods. In not, I would suggest removing the reference to ‘linear’ and the line fits in S2_Figure, since the spearman rank correlation characterizes the monotonic relationship between two variables, which is less restrictive than assuming a linear relationship.

Response to the Reviewer: We would like to thank the reviewer for the very valuable input. The reviewer is correct; we used Spearman’s rank correlation. So, we deleted passages which referred to a “linear” behaviour. As the reviewer is also questioning the hypothesis formulated in this paragraph, we decided to delete it completely. We agree with the reviewer that this hypothesis was a bit too motivated and was partly based on the results including one participant, which we excluded in the course of the previous review. We further deleted the lines in S2_Fig. which did suggest that we tested for a linear relationship.

Lines 278-280, suggest revising to read, “The level of significance for correlations was corrected to p < .0036 to account for testing across the coronal and sagittal plane CGA and MIM parameters.”

Response to the reviewer: We would like to thank the reviewer for this suggestion, which we changed accordingly in the new manuscript (now Lines 272-274)

Lines 293 & 297, I believe the table being referenced is Table 2.

Response to the reviewer: Correct! We apologise for this mistake, and changed the reference to table 2.

Line 336, the labels (and definitions) for FS, oFO, oFS, and FO are not in the revised figure and can be removed.

Response to the reviewer: The abbreviations for the discrete gait events are still present in the figure. They may not be as obvious as before, because we added the sub-phases underneath. So, we did not change the caption of the figure.

Line 376 references S2_Fig when stating that “…pelvic drop of the none-involved side was not present in our TFA group…”. Please clarify how S2_Fig shows this.

Response to the reviewer: We would like to thank the reviewer for noticing. This is a mistake and it should say “Fig. 2” not “S2 Fig.”. This part of the discussion was further rewritten.

Lines 451-0452, suggest revising to read, “For example, the affected side ground contact in a person with TFA is different than in an unimpaired person.”

Response to the reviewer: Thank you. We changed this sentence to: “Moreover, the affected side ground contact in a person with TFA is different than in an unimpaired person.” (Now in line 416-417)

Lines 454-455, the phrase “adapt the introduced”, seems incomplete. Suggest revising.

Response to the reviewer: Thank you. We revised this sentence to: “Thus, gait deviations in this population may be a response to unwanted loads introduced to the involved side, and may serve as an option to readjust loads.” (Now in line 419-421)

Lines 455-457, suggest revising to read, “Another indicator that coronal plane gait deviations cannot purely be attributed to hip strength deficits is that a lateral trunk lean can also present in amputations below the knee.”

Response to the reviewer: Thank you. We changed this sentence according to your suggestion (Now in line 421-422).

Lines 460-461, the phrase “…the reasons behind the strength deficits and the commonly, to strength deficits attributed gait deviations…” seems incomplete. Suggest revising.

Response to the reviewer: Thank you. Yes, this sentence was incomplete and we revised it to: “Hence, the origins for strength deficits in people with TFA and their gait deviations, partially attributed to these strength deficits, cannot be fully clarified in this study.” (now in line 425-426)

Line 468, suggest revising to read, “may induce a weakening of certain muscle groups…”

Response to the reviewer: Changed

Line 491, suggest revising to read, “participants reported being satisfied with…”

Response to the reviewer: Changed

Lines 507-509 state that “Over the course of this study, an additional hypothesis was developed, i.e. that people with TFA should not have a maximum hip moment below a certain threshold to avoid gait deviations.”. There does not appear to be any indication of a threshold effect in the results. Please clarify how the current data/analyses directly test this hypothesis.

Response to the reviewer: We deleted this paragraph completely. As stated earlier, we agree with the reviewer that this hypothesis was a bit too motivated and was partly based on the results including one participant, which we excluded in the course of the previous review.

Lines 515-518, suggest revising to read, “However, during walking compensation mechanisms may also be induced, e.g. by the socket, prosthetic leg length or prosthetic component related, that contribute to the net functional deficit.”

Response to the reviewer: Thank you. We changed this sentence according to your suggestion (now in line 470-471)

Other minor comments and suggested edits (insertions/deletions in brackets),
Line 80, “…reduced bone density and also [] volume loss…”

Response to the reviewer: Changed
Line 97, “Larger amounts of fat embedded in the residual limb[] muscles…”

Response to the reviewer: Changed
Line 174, “…aids differ[red] considerably from…”

Response to the reviewer: Changed
Line 290, “For temporal spatial parameters[,] individuals…”

Response to the reviewer: Changed
Line 294, “For CGA kinematic results[,] participants with…”

Response to the reviewer: Changed
Line 339, “…extension, and flexion) in [] both…”

Response to the reviewer: Changed
Line 340, “For hip abduction and adduction[,] MIMs in individuals…”

Response to the reviewer: Changed
Line 372-373, “However, the patter[n] described by…”

Response to the reviewer: Changed
Line 376, “…pelvic drop of the non[]-involved side…”

Response to the reviewer: Changed
Line 378, “…described a different patter[n] in a different pathology…”

Response to the reviewer: Changed
Line 381, “…with TFA show[,] in contrast to Trendelenburg[,] a coronal plane…”

Response to the reviewer: Changed
Line 389, “Although not clearly detailed[,] this muscle…”

Response to the reviewer: Changed
Line 415, “…individuals with TFA)[,] we observed…”

Response to the reviewer: Changed
Line 443, “…it may be advisable to [guarantee] passive

Response to the reviewer: Apologies for overseeing the wrong autocorrect result. We changed this.
Line 480, “…has negative impact on[] the preferred…”

Response to the reviewer: Changed
Line 481, “Consequentially, there seem[s] to be…”

Response to the reviewer: Changed

Reviewer #2: First off, I would like to thank the authors for taking time to improve their manuscript. This has really shown through in the introduction which has a better flow than in the previous version. My comments are mostly dedicated to updating the phrasing throughout the manuscript and restructuring of the discussion to make the manuscript more concise and link similar ideas together.

Response to the reviewer: We would like to thank the reviewer for the inspiring words. We also like to thank the reviewer for invested time and effort.

Minor:
Abstract:
Line 37: please update “convenience” to “convenient”

Response to the reviewer: Changed

Line 45: please update “abduction, adduction[,] extension[,] and flexion” with the commas suggested

Response to the reviewer: Changed

Line 56: consider updating “with significantly higher ranges of motion on the involved side” to “with significantly higher ranges of motion during involved side stance phase”. I suggest adding “stance phase” to the sentence as the trunk is not a part of the involved side.

Response to the reviewer: We like to thank the reviver for this valuable suggestion. We agree to this remark and changed the sentence accordingly (now in line 54-55)

Introduction:
Line 81: Delete “, as described by Probsting et al.” as this is implicit with the citation.

Response to the reviewer: Deleted

Line 87: Change “conversely” to “furthermore” as the authors are adding to the argument established in the previous sentence.

Response to the reviewer: Changed

Lines 97-99: Consider deleting the last sentence of this paragraph as it distracts from the points that the author has established about muscle strength and atrophy.

Response to the reviewer: We partly disagree with the reviewer and didn’t delete this. We have rewritten the sentences as the fatty degeneration is part of the structural change and muscle atrophy of the residual limb (now in line 94-97).

Lines 107-112: Consider updating: “In contrast to this, a more rigid fixation of the residual limb with the prosthesis, i.e. by osseointegration, or bone anchored prostheses, in individuals with TFA may have a positive effect on muscle strength. This hypothesis was supported in a study by Leijendekkers et al. who measured muscle volume. In the monitored subject with TFA the hip abductor muscle volume increased in the sound limb by 5.5% and in the residual limb by 7.4% during 12 months after implanting the bone-anchored prosthesis [16].”
to:
“In contrast to this, a more rigid fixation of the residual limb with the prosthesis, i.e. by osseointegration, or bone anchored prostheses, in individuals with TFA can have a positive effect on muscle strength [16].”

Response to the reviewer: Thank you for the valuable suggestion which wraps up the information very well. We have changed this accordingly (now in line 104-107).

Methods:
The methods overall read nicely.

Response to the reviewer: Thank you very much!

Page 11: Thank you for including more information regarding you TFA population and the procedure for prosthetic/socket fit.

Response to the reviewer: We appreciate that the reviewer is recognising that such details are important to us. Thank you!

Line 219: Consider deleting “following Kadaba et al. and Davis et al.” as this information is included in the citations.

Response to the reviewer: Deleted

Line 224: “was” to “were”

Response to the reviewer: Changed

Line 230: consider adding the following commas to the sentence: “The OpTIMo device[,] used to determine isometric hip moment[s][,] consist of a rigid frame….”

Response to the reviewer: Changed

Lines 278-279: consider deleting “as well” as it makes the sentence clunky

Response to the reviewer: Thank you for the suggestion. This shortcoming was also recognized by the 1^st^ reviewer and we changed the sentence according to his suggestion.

Results:
The results read nicely.

Response to the reviewer: Thank you!

Line 290: add a comma between “parameters” and “individuals”

Response to the reviewer: Changed. This was also identified by the 1^st^ reviewer.

Line 294: add a comma between “results” and “participants”

Response to the reviewer: Changed. This was also identified by the 1^st^ reviewer.

Discussion:
The following are recommendation to make the discussion more concise and to bring together similar ideas that were throughout the discussion.

Response to the reviewer: Thank you, we appreciate your suggestions.

Line 356: consider deleting “TTA” and updating the sentence to “persons with TFA” because it does not make sense to connect the results with persons with TTA as they are a completely different population.

Response to the reviewer: Deleted.

Lines 357-361: consider deleting these two sentences as it has you have already summarized these results in the previous sentence.

Response to the reviewer: We agree with the reviewer and deleted the partly redundant information.

Line 362-363: Why do the authors think that there was no strength deficit in hip extension as there were deficits the other directions? The authors have an explanation for this later in the discussion. I would recommend that they move lines 472-482 to the end of this paragraph.

Response to the reviewer: We agree with the reviewer and moved the sentence accordingly.

Please update some of these sentences as to not use the [author] et al…. (explanation). Updating the sentence structure will make the sentences clear and concise.

Response to the reviewer: We tried to delete most of the criticised sentences.

Line 367: consider updating “range” to “range-of-motion”

Response to the reviewer: Changed

Lines 371-378: Consider deleting these sentences as they do not add meaningful content to the author’s discussion as these sentences are dedicated to a population with a different pathology – which is stated in the last sentence fragment.

Response to the reviewer: We partly agree with the reviewer, that this is partly an off-topic. However, the pattern seen in people with TFA is often described as a Trendelenburg gait pattern, in particular in our community. We try to emphasis with these sentences that this is actually not correct. We deleted redundant parts of this section and tried to make it more concise. We hope that the reviewer is accepting our changes (see lines 359 following)

Line 369: Consider adding the following to the end of the sentence: “, as has been observed in people with TFA [24,25,42,45]”. The authors can then delete the sentence from lines 379-380.

Response to the reviewer: Thank you for the suggestion. We changed this accordingly (now in line 356-358)

Lines 383-391: It is suggested not to use the phrasing: [author] et al. showed….. this style of writing makes these sentences difficult to read. This information may be able to be summarized concisely. For example: “Altered trunk and hip kinematics exhibited in persons with TFA have been attributed to weak hip musculature [13,24,42].”

Response to the reviewer: Thank you for the suggestion. We tried to take this into account. The paragraph was changed accordingly (now in line 366-367).

The authors could then move lines 398-403 after this statement as it connects very well.

Response to the reviewer: We moved the section as suggested.

In my opinion, the authors should have the caveat in this paragraph that such a correlation was not observed in the healthy population. The S2 Figure (which I like) shows that less strength in the REF group would have a reduced trunk obliquity (p=0.02).

For instance: (the following is a suggestion for the authors, linking these statements together eliminates the reader having to go back and forth between paragraphs separated by another paragraph)
Altered trunk and hip kinematics exhibited in persons with TFA have been attributed to weak hip musculature [13,24,42]. This connection may explain the observed, non-significant, moderate negative correlation between hip abduction MIM and trunk obliquity range-of-motion. This correlation indicates that weaker people with TFA have the tendency to show a greater trunk range-of-motion during walking than their stronger piers. However, it should be noted that this is the exact opposite trend that was observed in the healthy population (S2 Fig.).

Response to the reviewer: We absolutely agree with the reviewer changed the section as suggested. This was very helpful and will improve readability. Thank you!

This may lead to lines 403-406 may be able to be appended (with some editing) to the paragraph from lines 392-397.

Response to the reviewer: Changed, shortened and edited accordingly.

Lines 409-410: consider update the start of the sentence to: “In typically developing children, the hip MIM…”

Response to the reviewer: Changed

Line 426: consider updating “contradictable” to “difficult”

Response to the reviewer: Changed

Lines 441-443: This sentence is very similar to the previous. It is recommended to end the sentence on line 441 with two citations [44,45] to eliminate the redundancy. I recommend then moving lines 445-447 after this sentence because it directly connects.
For example:
“For example, it is well accepted that quadrilateral sockets do not stabilize the femur in the coronal plane and therefore will more likely cause increased residual limb movement during walking, compared to other designs, e.g. ischial containment sockets [44, 45]. Decreased medio-lateral stability residuum and the femur, as a cause for a lateral trunk motion, along with other cofounding factors, like strength deficits of the hip abductors the socket [46]. The strength status of people with TFA is therefore of high clinical relevance.”
This recommendation does remove “So, in socket design it may be advisable to grantee passive stability and a very good force transmission, while not restricting the remaining muscles.” however, the proposed change does capture the importance of socket design.

Response to the reviewer: We would like toWe took over most of the suggestions of the reviewer.

499: “K_level” to “K-level”

Response to the reviewer: Changes

Conclusion:
Looks good!

Response to the reviewer: Thank you!

Reviewer #3: The authors made significant improvements to the manuscript. Overall, the writing is better and the authors have addressed my and other reviewer's comments. There a few minor issues to revise before being ready for publication.

Response to the reviewer: We thank the reviewer for these kind words. We also acknowledge the efforts und time of the reviewer. Thank you!

Slight formatting issues with table 1, column width is too narrow.

Response to the reviewer: We adapted this. We guess that such issues will be taken care of by the editorial office and the formatting of the tables will be adjusted to fit to their layout.
Line 28: I am assuming you mean mass, not weight.

Response to the reviewer: We apologize, but we do not know exactly which line the reviewer was referring to. We searched the document and changed weight to mass.

Line 443: grantee, incorrect word?

Response to the reviewer: Apologies for the typo, this supposed to say “guarantee”. This section was shortened and rewritten and this part was deleted.
Repeated use of consequentially, a different word or phrase in few instances to improve readability

Response to the reviewer: Yes, we seem to like this word too much. We changed the manuscript accordingly.
Check for consistent spacing after periods, fluctuates between 0 to 2 spaces

Response to the reviewer: We checked spacing.
Check - versus _ in regards to K-levels (e.g., lines 498-499)

Response to the reviewer: Changed

Thank you for addressing our comments.

Response to the reviewer: Your comments are appreciated and help to improve the manuscript considerably.
