# Supplementary material for: The influence of hip muscle strength on gait in individuals with a unilateral transfemoral amputation
Source: PLoS One. 2020 Sep 2;15(9):e0238093. doi: 10.1371/journal.pone.0238093 (PMC7467296; doi:10.1371/journal.pone.0238093)
Supplement: Attachment — Submitted filename: Response to Reviewers.docx [file pone.0238093.s010.docx]

Please use the space provided to explain your answers to the questions above. You may also include additional comments for the author, including concerns about dual publication, research ethics, or publication ethics. (Please upload your review as an attachment if it exceeds 20,000 characters)

Reviewer #1: (No Response)

Response from the Authors: We would like to thank the reviewer once again for the input. It helped considerably to increase the quality of the manuscript.

Reviewer #2: I would like to commend the authors on re-writing their manuscript. This manuscript represents a significant knowledge contribution in terms of how strength deficits can affect walking performance for persons with transfemoral amputation. Below I have listed some minor comments regarding word choice and punctuation.

Response from the Authors: We would like to thank reviewer for the positive words.

Line 67: “later” to “lateral”

Response from the Authors: Changed

Line 89: comma between “imaging” and “involved”

Response from the Authors: Changed

Line 90-91: suggest to update the sentence to: “This led to altered walking biomechanics and residual limb muscle activation patterns after a TTA [6]”

Response from the Authors: Changed

Line 118: “Beside” to “Besides”

Response from the Authors: Changed

Line 137: comma between “secondly” and “we”

Response from the Authors: We added a comma

Line 139: Replace semicolon with period and comma between “Finally” and “we”

Response from the Authors: Changed

line 147: comma between “data” and “maximum”

Response from the Authors: We added a comma

line 192: comma between “documentation” and “none”

Response from the Authors: We added a comma

line 220: consider updating the start of this sentence to: “As shown by Pamies-Vila et al., changes of the…”

Response from the Authors: We changed the start accordingly

line 242: comma between “measurements” and “a”

Response from the Authors: We added a comma

Discussion:
The flow of the discussion has improved greatly! Thank you for taking time and updating this section.

Response from the Authors: We would like to thank the reviewer for these positive words. This was also an effort of the reviewer. Thank you for helping to improve the manuscript.

Line 378: “will” to “would”

Response from the Authors: Changed

Line 378: comma between “gait” and “this”

Response from the Authors: We added a comma

line 386 “lead” to “led”

Response from the Authors: Changed

line 392: “conclude” to “concluded”

Response from the Authors: Changed

line 403: “transition” to “transmission”

Response from the Authors: Changed, we further updated the sentence as suggested.

Line 403-404: recommend updating the wording of this sentence to: “A reduced power transmission between the stump and the socket may cause the observed muscle weakness to manifest a more pronounced functional deficit.”

Response from the Authors: Thank you for this suggestion, we updated the sentence as suggested.

Line 406: “transition” to “transmission”

Response from the Authors: Changed

Line 412: comma between “discrepancy” and “was”

Reviewer #3: The authors addressed reviewers comments during this second review, so thank for continued improvements on this manuscript. Readability and clarity have been improved. There are a few more minor changes to be made:

Response from the Authors: We also thank the reviewers for their time and effort. Without their help we would not come to this stage of the manuscript.
Abstract:
Line 64: "i.e. later[al] trunk"
Line 68: "between later[al]"

Response from the Authors: Changed. Apologies for these typos
Introduction:
Line 92: "ultrasound imaging[,] involved"

Response from the Authors: Changed
Line 149: "movement; [f]inally we"

Response from the Authors: We changed this sentence in accordance to the suggestion of reviewer 2.

Methods:
Looks good, no suggested changes.

Response from the Authors: Thank you.
Results:
Looks good, no suggested changes.

Response from the Authors: Thank you.
Discussion:
Line 357: "First and foremost" is colloquial, could say "Our data shows that..."

Response from the Authors: We would like to thank the reviewer for that suggestion and changed the sentence accordingly.
Line 443: "it was conclud[ed]..."

Response from the Authors: Changed
Line 464: Remove 'A' at start of "A decreased..."

Response from the Authors: Changed
Line 465: Remove comma after "factors"

Response from the Authors: Deleted
Line 466: Add comma after "discrepancy"

Response from the Authors: Added
Conclusion:
Looks good, no suggested changes.

Response from the Authors: Thank you!
Thank you again for your continued work on this manuscript!Response from the Authors: It was our pleasure. Thanks
